# Cancer Disparities among Pacific Islanders: A Review of Sociocultural Determinants of Health in the Micronesian Region

**DOI:** 10.3390/cancers15051392

**Published:** 2023-02-22

**Authors:** Eric Pineda, Ryan Benavente, Megan Y. Gimmen, Nicole V. DeVille, Kekoa Taparra

**Affiliations:** 1 Tulane University School of Medicine, New Orleans, LA 70112, USA; 2 Harvard Medical School, Boston, MA 02115, USA; 3 Department of Epidemiology and Biostatistics, School of Public Health, University of Nevada, Las Vegas, NV 89154, USA; 4 Department of Radiation Oncology, Stanford University, Palo Alto, CA 94305, USA

**Keywords:** cancer disparities, Micronesia, Palau, Nauru, Kiribati, Mariana Islands, Guam, Pacific Islands, health disparities, Oceania

## Abstract

**Simple Summary:**

Micronesia is an ethnogeographic region within the continent of Oceania that represents an underserved area of the world. Cancer is the second leading cause of death among the people of Micronesia and indigenous Pacific Islanders share an unequal burden of the disease. Many factors come into play when discussing the adverse health outcomes of Micronesian people. Our paper intends to review these factors that have led to the ongoing health crisis among indigenous Pacific Islanders.

**Abstract:**

It is well appreciated that the social determinants of health are intimately related with health outcomes. However, there is a paucity of literature that explores these themes comprehensively for the indigenous people within Micronesia. Certain Micronesia-specific factors, such as transitions from traditional diets, the consumption of betel nut, and exposure to radiation from the nuclear bomb testing in the Marshall Islands, have predisposed certain Micronesian populations to an increased risk of developing a variety of malignancies. Furthermore, severe weather events and rising sea levels attributed to climate change threaten to compromise cancer care resources and displace entire Micronesian populations. The consequences of these risks are expected to increase the strain on the already challenged, disjointed, and burdened healthcare infrastructure in Micronesia, likely leading to more expenses in off-island referrals. A general shortage of Pacific Islander physicians within the workforce reduces the number of patients that can be seen, as well as the quality of culturally competent care that is delivered. In this narrative review, we comprehensively underscore the health disparities and cancer inequities faced by the underserved communities within Micronesia.

## 1. Introduction

### 1.1. Methods/Review Approach

Narrative reviews are a type of qualitative research that outline key results of scholarly work focusing on a topic of interest without focusing on the statistical significance of the findings [1,2]. We conducted a keyword search-based literature review using PubMed Advanced Search on or before 1 December 2022, for studies with abstracts or titles containing terms such as “Micronesia”, “Guam”, “Mariana”, “risk factors”, “social determinants of health”, “healthcare”, “diet”, “radiation”, “cancer”, and “oncology”. We limited this review to research on human subjects only and included English language-based peer-reviewed articles (e.g., primary research, reviews, and editorials), online reports, electronic books, and press releases. Based on the available studies at the beginning of this search, we focused our review on studies performed in the US, Micronesia, Australia, and New Zealand. All publications identified for review were assessed for relevance by the team of authors. 

### 1.2. An Introduction to Pacific Islanders and Micronesia

**Figure 1:**
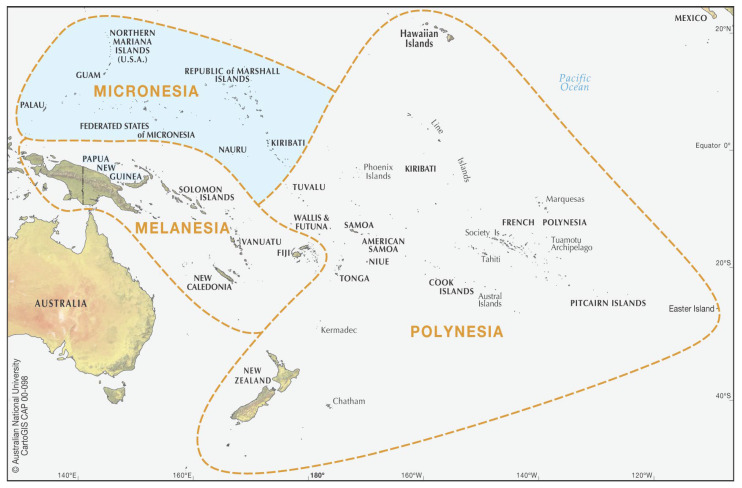
The three ethnogeographic regions from which Pacific Islanders trace their ancestry throughout the continent of Oceania: Micronesia, Melanesia, and Polynesia. The shaded area in blue represents the region and collection of island nations within Micronesia. The collection of approximately 2000 islands throughout the Federated States of Micronesia, Guam, Kiribati, the Republic of Marshall Islands, Nauru, Northern Mariana Islands, and Palau. Map provided by CartoGIS Services, Scholastic Information Services, The Australian National University.

**Figure 2:**
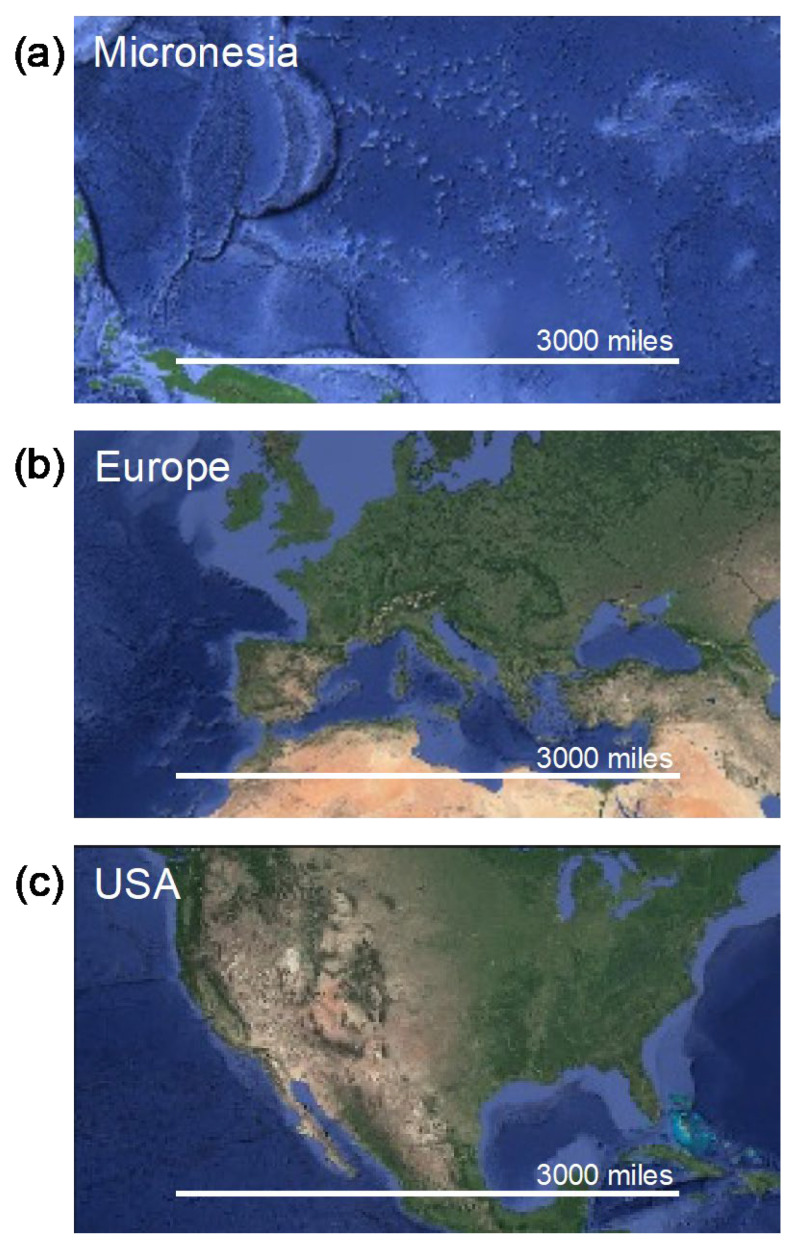
Satellite images displaying the relative size of (**a**) the Micronesian region within the continent of Oceania, (**b**) the continent of Europe, and (**c**) the United States of America. The white bar approximates 3000 miles to scale for all images. Adapted Google maps satellite images used with permission. Imagery ©2022 NASA, Terrametrics, Map Data ©2022.

The Pacific Ocean covers one-third of the planet’s surface [3]. Within the Pacific Ocean are the three ethnogeographic regions of the continent of Oceania from which Pacific Islanders share ancestry: Micronesia, Melanesia, and Polynesia (Figure 1). Together, these three regions comprise a collection of over 25,000 islands occupying over 300,000 square miles [3,4]. Each island population has its own distinct language, culture, and history [5]. Micronesia, more specifically, is comprised of over 2000 smaller islands and atolls dispersed across a region of the Pacific Ocean bordered by the Philippines to the West, Hawaii to the East, and Papua to the South (Figure 1 and Figure 2a). Micronesia is approximately 3000 miles across, comparable to the entire landmass of the US and the European continent (Figure 2). The island nations and territories in the Micronesian region include the Commonwealth of the Northern Mariana Islands (CNMI; a US commonwealth), Guam (a US territory within CNMI), the Republic of Belau (Palau), the Federated States of Micronesia (FSM; which consists of Yap, Pohnpei, Kosrae, and Chuuk), the Republic of the Marshall Islands (RMI), Nauru, and Kiribati [6]. Notably, the FSM, which is an island nation, should be distinguished from the ethnogeographic region of Micronesia, which encompasses all of these island nations and territories. Moreover, many of these Micronesian island nations and territories represent political groupings consisting of multiple islands and not single island nations.

The region of Micronesia encompasses nations and territories historically and politically tied to high-income countries (HICs) and low-middle income countries (LMICs). Independent islands, including Nauru and Kiribati, have ties to Australia and New Zealand, which represent LMICs. Five islands within the region of Micronesia have international relationships with the US and are referred to as US-Affiliated Pacific Islands (USAPI) and are naturally classified as having ties to HICs [7]. The current state of Micronesian international relations is deeply rooted in military strategy with a colonization history of Spain, Germany, Japan, and the US since World War I [8]. Following World War I, in 1947, the United Nations granted the US trusteeship over RMI, Micronesia, CNMI, and Palau, which allowed for subsequent US military occupation and weapons testing throughout the islands [9]. Decades later in 1986, a joint resolution known as the Compact of Free Association Act (COFA) established a relationship between these Freely Associated States (RMI, FSM, and Palau) and the US to help these Pacific Islands advance their economic development and self-sufficiency [10,11,12]. COFA granted the continued exclusive and strategic US military control over a large region of the Pacific; in return the USAPI were entitled to US security guarantees, disaster relief, health coverage, and economic assistance [11]. Political and economic affiliations to HICs or LMICs continue to have significant implications for health care resources and, more broadly, the local economy.

### 1.3. Cancer Disparities in Micronesia

Over the last half century, indigenous populations within USAPI have had dramatic shifts in health outcomes with some of the highest incidences in the world of preventable chronic diseases [13,14,15]. Cancer remains the second most common cause of death in nearly all USAPI nations. The sociocultural determinants of health are intimately intertwined with the cancer disparities that Pacific Islanders face in Micronesia. The historical trauma from nuclear weapons testing has led to generations of radiation-related diseases impacting both physical and mental wellbeing. Cultural practices, such as betel nut chewing, also remain an ongoing problem particularly among the indigenous populations of Micronesia, which contributes to cancer disparities among patients with oral cavity cancer. Transitions from traditional dietary behaviors, exacerbated by sedentary lifestyles, have led to the highest rates of obesity in the world, which have known associations with developing other chronic conditions such as cancer. 

The incidence of cancer in Micronesia is particularly staggering with the indigenous population experiencing an unequal burden of the disease [16,17]. The Pacific Regional Central Cancer Registry report cancer incidence rates (per 100,000 people) in USAPI from 2007–2018 [18]. Lung and bronchus, breast, invasive cervical, tobacco-related oral cavity and pharynx, liver, and prostate cancers are among the most common cancers affecting the people of Micronesia (Table 1). The incidence of cervical cancer is as high as 65.8 in RMI and 40.7 in Pohnpei, 5–8-times higher than the rates in the US. Similarly, incidence rates of oral cavity and pharynx cancer are 4-times higher in Yap, and rates of liver cancer are 2–3-times higher in Palau and Yap, compared to rates in the US (Table 1). For cancers of the lung, we see some of the lowest 5-year survival rates ranging from 4% in Yap to 30% in Guam. Breast cancers, which typically have higher survival rates, are lower in places such as RMI and Pohnpei, with many of the cancers being detected at stage 3 or later (Table 1). The incidence of nasopharyngeal cancers in Micronesian men is up to 4-times higher compared to men in the US state of Hawaii [16]. Given these cancer disparities, throughout this narrative review, we aim to underscore the multitude of factors that influence the significant cancer disparities observed across Micronesia.

## 2. The Social Determinants of Health in Micronesia

### 2.1. Social Determinants of Health

**Figure 3:**
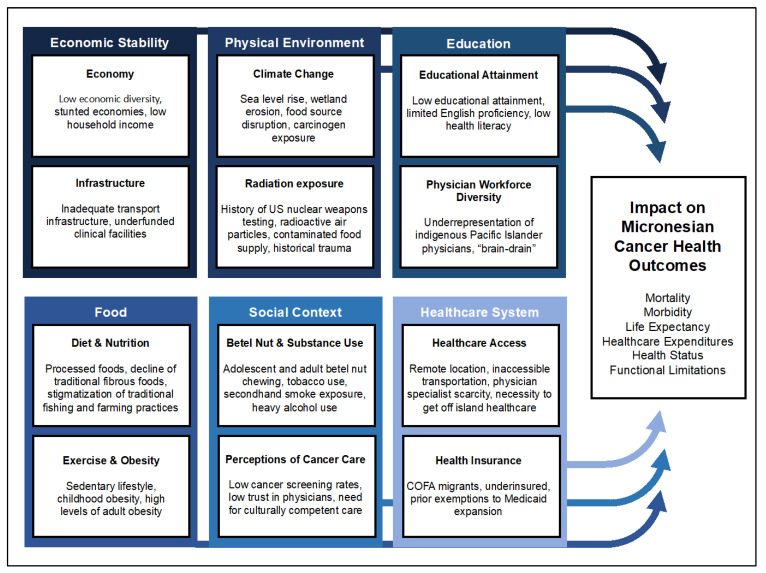
Conceptual framework of the social determinants of health as it pertains to Micronesian cancer disparities. Some of the known social determinants of health include economic stability, physical environment, education, food, social context, and the healthcare system.

The social determinants of health are essential social factors or conditions that directly or indirectly contribute to the development of disease among the general population including access to resources, power, prestige, and class [19]. The Kaiser Family Foundation defines Social Determinants of Health as the “conditions in which people are born, grow, live, work, and age” [20]. Cancer risk is irrevocably linked to the social conditions of diet, environmental exposure, substance use, education, physical activity, socioeconomic status, and the infrastructure of a country, affecting health outcomes, survivorship, and quality of life [21,22]. Hence, Micronesian-specific social determinants of health are important to discuss, as they can alter the course of an individual’s prognosis, may provide an explanation for disparate rates of certain cancers, and are an important source of knowledge to improve cancer interventions [22]. While social determinants of health vary widely among and between island nations and territories across Micronesia, the purpose of this review is to highlight general themes within the literature that underscore the health-related disparities that may influence the observed cancer disparities throughout the region. Throughout this review, we will touch on aspects of Micronesian Cancer Disparities, as they pertain to the conceptual framework of the social determinants of health (Figure 3).

### 2.2. Diet and Nutrition

Dietary factors have a significant impact on overall cancer incidence [23]. For example, a large meta-analysis of 45 observational studies demonstrated that lower colorectal cancer risk was associated with higher intake of fiber, dietary calcium, and yogurt [23]. Historically, Indigenous Pacific Islanders had incredibly diverse agroecosystems that were cultivated in sustainable practices and had the ability to support hundreds of thousands of individuals on some of the most geographically remote islands in the world [24]. Pacific Island communities traditionally adopted variations on a common diet depending on the produce, fish, and fauna that were accessible. For example, within the FSM, bananas, taro, copra, and breadfruit are considered staple foods and are incorporated into local cuisine [25]. This is due in part to the fact that the majority of accessible produce is optimally grown within the tropical Micronesian climate. For protein, Pacific Islanders traditionally had access to locally sourced poultry, fish, crustaceans, and mollusks; pork has often been reserved for special occasions. Tropical fruits, such as mango, guava, sour-sop, papaya, and pineapple, are also widely available between islands [26].

As a result of colonization and globalization, modern-day supply chains connected to the Micronesian Islands have increased the import of more refined grains and processed foods from overseas such as white rice, high fructose corn syrup products, and red meats [27]. Over the past century, many of these foreign imports have slowly replaced traditional Pacific Islander diets due to availability, affordability, and convenience [28]. For example, due to the influence of Japanese imperialist occupation, rice has become an integral part of the modern FSM diet [27]. One participatory mixed methods study including nearly 300 adult Pohnpeian women used a 7-day food frequency questionnaire to assess diet and found that 96% of participants reported eating rice frequently (3–7 days/week); only 75% consumed locally grown carbohydrates as a part of their normal diet [28]. A shift from foods that are imported, with low nutritional value, back to indigenous, high fiber, healthier foods require culturally sensitive and innovative strategies to promote local and sustainable food production.

The introduction of the Western diet with US influence has also drastically changed the food landscape and dietary behaviors among the people of Micronesia [27]. Given the remoteness of the Pacific Islands, historically, many foods imported into the islands had to withstand long distances of travel, necessitating foods and goods that were highly processed. These canned and highly processed food products are widely consumed among Pacific Islanders and may have some level of carcinogenicity, with several studies demonstrating increased risk of stomach and colorectal cancers [29,30]. In 2015, the International Agency for Research on Cancer Working Group made a formal recommendation that the consumption of processed meat be classified as a Group 1 carcinogen, labeling these products as “carcinogenic to humans” given associations with colorectal and gastric cancer [29]. This inequity in food accessibility is exacerbated by a culture of misinformation, as the giant swamp taro, once widely cultivated and consumed by Micronesians, is in some cases shunned for being “starchy” and is instead being fed to pigs and other farm animals [25]. Vegetable and fruit consumption is also disproportionally low throughout Micronesia. For example, over 80% of the Pohnpei population consume less than five servings of fruits and vegetables per day with an average of 1–2 servings per day, while in Majuro 40% and 23% of youth were found to not eat a single serving of “a green salad” or “fruit” at least once per week, respectively [13].

Sociocultural factors also influence the diet of Micronesians in the Pacific Islands. Imported food are perceived as “higher quality,” which gives these items a level of societal “prestige” [31]. Conversely, in some Micronesian communities, the practice of farming is becoming culturally stigmatized. For example, in the Cook Islands, those who farm are called “Repo Taro” or “Dirty Taro” [32]. This derogatory term is used to refer to men who perform manual labor (e.g., taro farming) and are stigmatized as men who “are nothing” and “have nothing”, such as money, land, or other capital [33]. Simultaneously, traditional knowledge and practices of food cultivation, preservation, and farming are being lost with each subsequent generation [31]. As a result, local subsistence agriculture and fishing have all steadily decreased over time, leading to increased economic costs to families and to Pacific Island nations [32,34]. Poverty and a growing reliance on government food subsidies has also contributed to the loss of farming and maintenance practices for many indigenous crops, such as taro, arrowroot, breadfruit, and other long storage crops [32]. This is exacerbated by some islands lacking refrigerators, as low as only 10% of households in more geographically isolated islands, such as Pingelap, which creates greater dependence on canned foods [35,36].

### 2.3. Exercise and Obesity

Physical activity, even if only leisurely, has demonstrated significant benefit to overall health and has been associated with a decreased risk for both cardiovascular and cancer mortality [37]. A large meta-analysis demonstrated that obesity is associated with worse overall and cancer-specific mortality among patients with breast and colorectal cancer [38]. Globally, the top 10 countries with the highest rates of obesity are all Pacific Island nations, including those within Micronesia [39]. In many islands, including Pohnpei and Kosrae, over 80% of the adult population are obese [31]. This observation applies outside of the geographical region of Micronesia, as a study conducted in Hawaii demonstrated that ethnically Pacific Islander children are nine-times more likely to be overweight or obese when compared to White children [40]. 

However, in Micronesia today, physical activity and exercise rates are low among the general population, with most Pacific Islanders failing to vigorously exercise 3-times a week, moderately exercise 5-times a week, or achieve over 600 metabolic equivalents per week [41,42]. For example, over 60% of residents of Chuuk reported low levels of daily physical activity with lifestyles centered around sedentary activities instead (e.g., “talking story” and weaving) [41]. Weight management and exercise implementation programs are rare and largely non-established in Micronesia [43]. Dietary factors, coupled with cultural beliefs, a lack of exercise, and a sedentary lifestyle, have led to an increase in non-communicable diseases, such as diabetes, hypertension, and obesity, as well as deficiencies in essential vitamins and nutrients [25,43]. These comorbidities have been shown to be associated with an increased risk for several different types of cancer, as well as other adverse health outcomes [44,45,46,47,48,49]. 

### 2.4. The Economy and Infrastructure

Socioeconomic status is fundamentally linked to health outcomes [19]. In the US, patients with cancer who live in counties with lower income are associated with higher cancer-specific mortality [50]. Within Micronesia, the majority of independent states are classified as low-income countries with GDPs far below the world average, with among the slowest GDP growth globally and heavy dependence on US aid [51,52]. Poverty rates and resource access differ between communities. For example, in the CNMI, due to a decrease in tourism, garment industry closures, and the rising cost of fuel, over half of Micronesians live below the poverty line, and more than 1800 families make less than $5000 US dollars annually as recently as the 2010s, given a total population of approximately 50,000 [51,53,54]. Whereas in Palau, 25% of people live below the US poverty line, and in the FSM, 15% of people live on less than $1.90 per day [51]. Opportunities for work are predominantly in government or tourism industries, while growing overreliance on imported goods and services further stunts the growth of the economy [51,52]. A shift away from the agricultural industry, due in part to the perceived undesirability of the career and wage differential compared to other sectors of the workforce, has further increased the economic challenges these islands face [55]. These challenges have reportedly been associated with an alarming increase in the rates of drug use and reported suicides, especially among youths [55].

It is well appreciated that developing countries tend to have worse cancer mortality due in part to the lack of healthcare access, limited infrastructure, and low availability of cancer screening [56]. These disparities are apparent throughout Micronesia. For example, in Chuuk, water is primarily gathered from rain run-off, while cleaning facilities are rated as poor by members of the community [55]. Some of the outer islands do not have consistent access to power, relying on kerosene. These are invaluable resources that are necessary to the infrastructure of a functioning healthcare system, let alone a specialized cancer center. Despite efforts to urbanize and improve infrastructure, most of the islands within the FSM do not have paved roads, making it difficult to travel to nearby community health centers [13]. These structural factors put a significant strain on Pacific Islander patients and abrogate access to the most essential healthcare. Beyond the accessibility of these facilities, many of the island’s clinics are limited in the scope of healthcare they can offer due to underequipped and aging facilities [57]. Overall, the state of healthcare access throughout Micronesia is limited and impacts the overall health and wellbeing of the indigenous people who reside in these islands.

### 2.5. Educational Attainment among Micronesians

In developed countries, such as the US, there is a well-known association between lower educational attainment and higher mortality [58]. Since 1998, for patients with cancer, there has been an increasing gap in cancer-specific mortality between the least and most educated patients [59]. Throughout Micronesia, educational and vocational attainment varies across islands. In Palau, 92% of the country is literate, and more than 75% of men and 82% of women have attended some college education [53]. In comparison, a report in 2013 showed 43% of CNMI adults aged 25 and older have completed high school, while only 18% of FSM Micronesians attended college [60,61]. Furthermore, most of the FSM have limited English proficiency. Barriers to educational attainment include a lack of effective policy framework, a developing educational and economic infrastructure, financial constraints, and the lack of a culturally appropriate “Western education” [62]. Lower educational attainment is linked to worse health outcomes, limited economic mobility, as well as adverse risk behaviors (e.g., substance use, tobacco smoking) [63,64]. This is not just limited to Micronesians living in Micronesia. In Hawaii, between 2020–2021, 59% of Micronesian students were unable to pass their high school mathematics classes [65]. Given an individual’s level of education is associated with health literacy and the ability to navigate the healthcare system and use of primary care services, improving educational equity among Micronesians may provide a steppingstone towards improved cancer outcomes and health equity in general [66].

### 2.6. Summary of Diet, Exercise, Economy, and Education in Micronesia

In this section, we reviewed some of the major social determinants of health as they pertain to Micronesian patients in the setting of cancer. This section included diet and nutrition, exercise and obesity, economy and infrastructure, and educational attainment. Table 2 highlights critical areas of each of these factors along with possible solutions moving forward.

## 3. Exposure to Radiation and Substances

### 3.1. Radiation Exposure in Micronesia

Historical nuclear weapons testing by the US government in the Marshall Islands from 1946–1958 has led to the deposition of radioactive materials in the air, water, and land [67,68,69]. Many studies have demonstrated the associated increased risk of developing cancer with exposure to the radioiodine fallout following nuclear bomb testing in the Pacific, particularly cancers of thyroid origin [67,68,70]. Although a mass evacuation was orchestrated by the US government, islanders from the CNMI were found to have radionuclides within their urine following atomic bomb testing in the Marshall Islands [71]. Furthermore, despite the discontinuation of these tests several decades ago, radioactive particles remain in significant amounts and are still distributed by winds to populated areas [72,73]. Some studies have suggested this phenomenon has contributed to the increased risk of acquiring thyroid and hematological cancers by 20% and 5%, respectively [72,73]. 

The risk of radiation exposure varies by island, with those closest to the detonations or downwind of the detonations having more exposure than others [72,73]. Residents of the Marshall Islands born in the 1950–1970s (the decades during or directly following radioactive testing) were also at increased risk given chronic exposure to greater concentrations of background radiation in-utero or as children/adults [72,73]. There is also an individual risk of radiation exposure due to contaminated food and water. For example, some fruits on Rongelap remain contaminated with levels of radionuclide Cesium−137 that far exceed safe consumption standards [72,73]. In addition to radiation exposure related to colonization and militarization, fish have been contaminated by polychlorinated biphenyl pollutants released by ships used for the nuclear test targeting [74]. 

The impact of nuclear bomb testing is still an area of great research interest, due to the lack of research and data on these populations immediately following the nuclear bomb testing. Although radiation has declined over the years, there is still an inherent risk of radiation exposure associated with living within the Marshall Islands [69,72,73]. For example, efforts to resettle the affected islands of Rongelap, Bikini atoll, and the various outer islands are currently unadvisable [75]. The increased levels of background radiation may continue to pose future problems to the ecosystem and people living in adjacent areas [69,72,73,75]. Years of trauma associated with radiation testing has led to generations of perceived radiation-related birth defects, cancers, and other chronic disease, which may explain in part why Pacific Islanders are particularly at risk of refusing potentially curative radiation therapy in the US [76,77].

### 3.2. Betel Nut and Substance Use

Consumption of the carcinogenic areca palm seed, better known as the betel nut, is a growing epidemic directly associated with the development of aggressive oral cavity cancers across the region within Micronesia [78,79,80]. The practice of areca nut chewing is also associated with an increased risk of metabolic disease, cardiovascular disease, and all-cause mortality [53]. The areca nut can be consumed simply by itself or in conjunction with slaked lime, tobacco, and/or the betel leaf (this combination is referred to as betel-quid) [81]. This sociocultural practice is commonly called “chewing betel nut.” Over half of the adult population in Micronesia report betel nut chewing, compared to the world average of 20% [79,82]. The 5-year areca nut chewing prevalence in Guam was 11% and increased among non-Chamorros, primarily other Micronesians, from 7% in 2011 to 13% in 2015. Nearly half of adult chewers in Guam preferred areca nut with betel leaf, slaked lime, and tobacco, while nearly half of youth chewers preferred areca nut only [79,83]. 

The World Health Organization reports that betel nut chewing is an epidemic particularly among Micronesian youths who have the highest rates of betel nut chewing in the world among school-aged children [84]. In Yap and Pohnpei, betel nut continues to be chewed by the majority of 7th and 8th graders, with 75% of Palauan high school students having tried betel nut at least once. The increased prevalence and adverse outcomes associated with oral cavity cancers and lesions can be attributed to high levels of betel nut consumption [85]. In Yap, oral cavity cancer has an incidence of 22 per 100,000, which is two-fold greater than the US national average of 11 per 100,000 [86,87]. In the CNMI, between 2005–2019, 79% of all head and neck cancers were oral cavity cancers, with those who used betel nut being diagnosed at younger ages (47 years versus 55 years) and with a decreased five-year survival rate of 50% [86,88].

In addition to the betel nut, tobacco is regularly co-consumed, increasing the risk of oral cavity and other related cancers [16,17]. Betel nut chewers co-consume tobacco at a rate of 90% and 80% in Pohnpei and Palau, respectively [17]. Most adult smokers in Pohnpei started smoking before the age of eleven [17]. Guam also has a high prevalence of current smokers at 25% [89]. Adolescents are at risk for these behaviors and exposure to secondhand smoke. There are reportedly high rates of adolescents who observe regular smoking in public places or have parents who smoke [13,90]. Likewise, although alcohol consumption varies between island populations, heavy drinking is another risk factor for cirrhosis, liver failure, and certain cancers [91]. For example, on Nauru, the Cook Islands, and Chuuk, men who consume alcohol are more likely to be specifically “heavy drinkers” defined as consuming more than six drinks per day [92]. This may be a possible explanation for the development of hepatocellular carcinoma seen in Micronesians [33]. When compared to other US born Pacific Islanders, Micronesians born outside of the US are more likely to be diagnosed at an earlier age of hepatocellular carcinoma (52 years versus 60 years) and have more tumors (64% versus 32%), often associated with hepatitis B and liver cirrhosis [93]. Overall, there are multiple highly prevalent addictive substances consumed within Micronesia that have direct implications for cancer incidence and clinical outcomes.

### 3.3. Summary of Micronesian Radiation Exposure and Substances

In this section, we reviewed two types of exposures that have been associated with cancer specific disparities among the Micronesian population. We reviewed both radiation exposure related to nuclear weapons testing, betel nut chewing, and the consumption of other substances. Table 3 highlights critical areas of each of these factors along with possible solutions moving forward.

## 4. Cancer Care in Micronesia

### 4.1. Health Care Access and Cancer Surveillance, Diagnostics, and Treatment

Compared to the integrated supply networks of continental landmasses that freely transfer goods and services from one region to another, the islands of Oceania are the most remote regions on the planet, which significantly limits their ability to transfer and access essential health care resources [5]. For example, Pohnpei is 70 km (43 miles) from the closest populated island, with nothing but open ocean between [6]. With over 600 islands across 18,000 miles in the FSM, just a few islands contribute to most of the country’s population [6]. Atolls are the most isolated landmasses, with often only a few families living on the entire island [35]. Many of the islands rely heavily on outside support and foreign aid, but the considerable distance from major shipping ports, airports, and cities adds difficulty in collecting medical supplies. Not only are the physical equipment and supplies in shortage, but many islands throughout the Pacific face significant staffing shortages due to emigration of physicians who leave practices on remote Pacific Islands for opportunities in the continental US or elsewhere [94]. These problems are more apparent in the outer islands of Micronesian nations, which depend on infrequent cargo shipments [13]. 

Like many developing countries, the healthcare infrastructure of Micronesia is underdeveloped. Many of the major islands have clinics and healthcare services, but the quality, variety, and level of specialty care varies between islands [57]. Furthermore, major hospitals are not always located in proximal islands, but rather are scattered throughout the Pacific. For example, the only major hospital in the entire US territory of the CNMI that provides primary care, dialysis services, disease screening tests, substance abuse treatment, mental health evaluations, and all public health services is located on the island of Saipan. In the RMI, the major hospitals are on the islands of Majuro and Ebenye, which serve twenty-four islands in the country. Not only are these hospitals scattered throughout the Pacific, but they are only accessible by boat or plane, which make emergency care for those living on atolls very difficult [57].

Cancer surveillance in Micronesia under current healthcare infrastructure varies depending on proximity and access, both physical and economic, to major hospitals. Most USAPI nations and territories, with the exception of FSM, have access to CDC breast and cervical cancer early detection programs, prostate cancer screening, and colonoscopy capabilities. Specific tests, such as transrectal ultrasound, are not available in certain locations, including CNMI and Palau (Table 4) [18]. In other cases, the island of Yap might have the equipment, such as a colonoscope, but at certain times of the year no specialist who is trained in performing the procedure, such as a board-certified gastroenterologist [57]. 

Cancer diagnosis and treatment is limited by availability of equipment and personnel. Many islands have available general surgeons and obstetricians and gynecologists, but most had a shortage of or complete absence of oncologist or urologist (Table 5) [18]. Guam was the only island with MRI and PET imaging capacity. Chemotherapy and radiation therapy was largely unavailable in most islands, except for Guam (Table 5) [18]. All of these factors have the potential to delay cancer diagnosis and treatment, limiting the options for Pacific Islanders who are already at high risk for developing chronic diseases, such as cancer. Given specialty care is inaccessible across many USAPI, expensive off-island referrals and medical evacuations have become normalized [95,96]. This poses a significant healthcare cost. For example, in ten years, Tuvalu in Polynesia spent 44% of its budget on outside medical referrals [96]. The total cost of medical referrals is estimated to be $125 million as of 2017 [96]. This is a major issue in healthcare delivery and equity, as the financial costs and geographical distance prevent many Pacific Islanders from receiving the care they need until it is too late [95,96].

### 4.2. Health Insurance

Insurance status is known to be associated with access to medical care, specialty services, and severity of health outcomes [97]. Many of the islands, including Chuuk and the Marshall Islands, have government plans for citizens to access regional health facilities within geographical proximity and even larger, more specialized Hawaii hospitals [13,14,15]. However, up until recently, most COFA migrants were underinsured due to being ineligible for Medicaid (US-sponsored insurance program for low-income individuals and families) [98]. Despite the Affordable Care Act of 2010 increasing health insurance coverage for most Americans in the 50 states, Micronesian migrants were one subgroup that were significantly disadvantaged by this policy and exempted from Hawaii coverage and access to specialty care, including cancer care [99,100]. This resulted in more uninsured emergency medicine department visits in 2015, the year that COFA migrant coverage expired [98,99,100]. Soon after from 2015–2018, mortality rates for COFA migrants concurrently increased by 21% and the overall healthcare utilization (net inpatient and emergency room visits) decreased [99,100]. If care is not being utilized appropriately due to a lack of insurance, greater risks of developing later stage cancer and worse health outcomes are to be expected due to decreased screenings and delayed treatment [101,102]. 

COFA healthcare coverage was reinstated in 2020, with the passage of the Consolidations Appropriations Act [103]. Despite changes in policy, COFA migrants and other Pacific Islanders were disproportionately affected by the COVID-19 pandemic [104,105]. Pacific Islanders in general, despite making up 4% of the population, made up 24% of the COVID cases in Hawaii [105], with people of Chuukese and Marshallese heritage comprising 24% and 22% of all Pacific Islander cases, respectively. Furthermore, mortality rates (319.6 per 100,000 people) far exceeded the mortality rates of other demographics [105]. For those with specific cancers or undergoing systemic therapy, COVID-19 can be especially deadly due to the immunocompromised state of patients with cancer undergoing chemothearpy [106]. This disparity highlights that the presence of health insurance, while necessary for good health outcomes, is not sufficient. More research measuring insurance coverage and barriers to obtaining healthcare among this underserved population is needed, although this poses a challenge since the US census does not directly disaggregate data on Micronesian ethnicity [107].

### 4.3. Perceptions of Cancer Care

Cultural beliefs also contribute to barriers in accessing preventative cancer screening. In a study using the Health Information National Trends Survey data, 63% of all Pacific Islanders over 50 years of age in Hawaii had not underwent routine cancer screening as recommended by a physician [108]. Further, Marshallese and Chuukese people in Hawaii are twice as likely to trust information from priests compared to information from doctors [108]. In other cases, there is a strong desire to be screened and receive periodic check-ups, but there is a lack of access in general. In Yap, only 28% of all cervical cancers are caught at stage 1, significantly less than the 56% of invasive cancers diagnosed in the US [57]. This may be due to low accessibility of urine and Pap screens within these islands, despite research showing that women in Yap express a preference to undergo recommended cancer screening [109]. Results from a pilot community-based participatory randomized control study of women in the FSM indicate that 95% of women were comfortable with urine tests, and 82% of women were comfortable with a Pap smear for cancer screening [109]. Overall, there is a discordance between people in Micronesia who want cancer screening and those who receive cancer screening.

### 4.4. Coalition Building in Micronesia

A need for a comprehensive approach to cancer management in Micronesia has led to innovative solutions [110,111]. In 1997, a regional, concerted approach was developed with a coalition of health leaders from USAPI to strengthen management of cancer care and prevention [111]. Funding from the US National Cancer Institute and National Institutes of Health led to the formation of the Pacific Regional Comprehensive Cancer Control Program and partners (PRCP) in 2002. Since its inception, a number of partners were established that enabled the development of a comprehensive cancer control program aimed at improving key areas such as collaboration and sharing of resources between islands, cancer management in children, point-of-care testing and treatment, and surgical services [110,111]. The PRCP regional central cancer registry was also formed, which allowed for systematic collection of cancer data in the region. Data collected since 2007 has already informed policy decisions and loco-regional health care practices [111]. 

### 4.5. Summary of Healthcare and Health Perceptions

In this section, we reviewed themes within healthcare including access, insurance, and perceptions related to cancer within Micronesia. Table 6 highlights critical areas of each of these factors along with possible solutions moving forward.

## 5. Climate Change in the Pacific

### 5.1. The Impact of Climate Change on Cancer and General Health

From the increase in environmental carcinogen exposures to disruptions to cancer care access, global warming and climate change directly impact the accessibility and quality of care for patients with cancer around the world [112]. Pacific Islanders are among those disproportionately affected and are in direct significant danger from the impeding threat of climate change. Small, low-elevation islands in Micronesia are in jeopardy due to sea level rise, risk of tsunami, flooding, and other adverse weather events, which threaten the livelihoods of small communities that inhabit remote islands throughout the Pacific [113]. On a global level, climate change has shown to negatively impact cancer treatments and clinical outcomes. In a study looking at over 1700 patients undergoing radiation therapy for non-operable non-small cell lung cancer during hurricane disasters, compared to propensity matched patients who underwent similar treatment without exposure to natural disasters, the group exposed to natural disasters had worse overall survival [94]. These findings suggest the need for effective public health research and sustainable interventions that will monitor and shape the health of small island populations predicted to be at high risk for adverse health effects due to climate change [114]. 

Compared to continental land masses, island topography tends to be close to sea level and thus low-lying islands, such as those in Kiribati and RMI, are susceptible to sea level rise, capable of rendering entire islands uninhabitable and impacting healthcare access [113]. Food insecurity, malnutrition, fresh water accessibility, and displacement due to climate change and sea level rise are real threats that can have a disproportionate burden on patients from marginalized communities, including those with cancer in Micronesia [115,116]. Severe weather events make it difficult to make medical appointments, face-to-face encounters, and routine cancer screenings. This was apparent in 2007, when an acute-onset sea level rise event occurred in two atoll islands of Micronesia, which resulted in extensive coastal erosion, shoreline inundation, and saltwater intrusion [114]. The impact of the sea level rise went beyond the coastal shores, but also led to disastrous losses of crop productivity and limitations of freshwater resources. Of a total of 112 households surveyed, representing nearly 1000 residents in Micronesian islands, nearly all families had a partial loss, and one in three households reported complete loss of critical farmed produce, including taro or breadfruit [114]. 

Even islands that have mountainous landscapes with high elevation are at risk for a rise in health problems over the coming decades, which will only increase the burden on an already challenged healthcare system [113]. For example, climate change has been associated with a rise in infectious vector borne diseases, heat effects, and malnutrition, which impacts patients from Micronesia [117]. The World Health Organization projects approximately 250,000 additional deaths per year by 2030 from malnutrition, diarrhea, and heat stress attributable to climate change [113]. These more global health impacts can certainly impact the overall health trajectory of Micronesian patients with cancer. Thus, islands throughout Micronesia are vulnerable to multiple types of climate change and natural disasters capable of stripping islanders from already limited resources essential for health and wellbeing. Overall, there is a great unmet need to better understand how climate change may impact cancer care throughout Micronesia to build more climate resilient health systems.

### 5.2. Summary of the Impact of Climate Change in Micronesia

In this section, we reviewed how climate change impacts health care and cancer care. We discussed how climate change, particularly with a focus on Micronesia, may impact cancer care. Table 7 highlights critical areas of each of these factors along with possible solutions moving forward.

## 6. Micronesia and Physician Workforce Representation

### 6.1. Review of Physician Workforce Diversity in Micronesia

In general, there is a shortage of physicians in Micronesia, with many islands including FSM, the RMI, Kiribati, and Vanuatu have less than two physicians per 1000 people [95]. This is due, in part, to multiple factors including the lack of medical training programs in Micronesia, requiring prospective physicians to go off-island for medical training. Many motivated Micronesians are unable to leave and face barriers to entry including prohibitive training costs, familial obligations, and a lack of social or familial support critical to success in medical training [118,119,120]. Furthermore, there is a phenomena of “brain-drain,” where highly-skilled physicians do not return to their home communities or are poorly retained, instead choosing to work in other areas, including New Zealand, Australia, and the US, resulting in a decreased density of doctors in certain areas [118,119,120]. This data underscores the need for more pipeline programs throughout Micronesia, and Oceania more broadly, with the support of government funding to bolster the next generation of physicians with ties to these islands to promote high-quality, accessible, and longitudinal care for their communities [121].

In the US, Pacific Islanders are among the least represented racial groups in medical schools and are significantly underrepresented throughout the physician pipeline [67,121,122]. The number of Pacific Islander medical students, residents, and academic faculty in the US appear to be significantly declining in the past twenty years [123]. Moreover, there is virtually no Pacific Islander representation within oncology in Micronesia, let alone throughout the US [67,123]. Increasing the representation of Micronesian healthcare providers allows for cultural connection and understanding between Micronesian patients and their physicians, which may strengthen the physician–patient relationship and improve healthcare delivery [124]. In the US, many Chuukese people have experienced a cultural disconnect with their physician, insensitive comments, or racial discrimination, which overall serve as barriers to health equity among Micronesians [125]. Improving representation of Pacific Islanders in medicine should be prioritized to promote health equity for Pacific Islander patients through culturally competent care.

### 6.2. Summary of Micronesian Physician Workforce Representation

In this section, we reviewed limitations to the Pacific Islander physician workforce representation in Micronesia and the importance of developing strong pipelines throughout Oceania to improve health equity for Micronesians. Table 8 highlights critical areas of each of these factors along with possible solutions moving forward.

## 7. Conclusions

Micronesia is a vast network of islands with unique healthcare needs. To promote a healthy Micronesian community, a thorough understanding of the social, cultural, and economic determinants of health is important. Tremendous innovation and work have already been accomplished in building coalitions to promote positive public health outcomes. Further research and attention to Pacific Islanders in Micronesia, and Oceania more broadly, is warranted to reduce cancer disparities and promote health equity.

## Figures and Tables

**Table 1:** Cancer incidence, 5-year survival, and % diagnosed late-stage based on the Pacific Regional Central Cancer Registry 2007–2018 data. Incidence rates are reported as incidence per 100,000 people, age-adjusted to the US standard population [18].

Top Three Most Frequent Cancers in USAPI, 2007–2018									
Guam	**Lung**			**Breast**			**Prostate**		
	Incidence	5-year Survival	Diagnosed Late-Stage	Incidence	5-year Survival	Diagnosed Late-Stage	Incidence	5-year Survival	Diagnosed Late-Stage
	52.6	30%	90%	82.1	91%	63%	84.6	88%	49%
Commonwealth of the Northern Mariana Islands	**Breast**			**Oropharynx**			**Lung**		
	Incidence	5-year Survival	Diagnosed Late-Stage	Incidence	5-year Survival	Diagnosed Late-Stage	Incidence	5-year Survival	Diagnosed Late-Stage
	34.5	90%	73%	9.7	58%	76%	16.8	38%	86%
Republic of Marshall Islands	**Cervical, Invasive**			**Lung**			**Breast**		
	Incidence	5-year Survival	Diagnosed Late-Stage	Incidence	5-year Survival	Diagnosed Late-Stage	Incidence	5-year Survival	Diagnosed Late-Stage
	65.8	57%	49%	31.4	8%	86%	23.3	72%	61%
Republic of Palau	**Lung**			**Liver**			**Prostate**		
	Incidence	5-year Survival	Diagnosed Late-Stage	Incidence	5-year Survival	Diagnosed Late-Stage	Incidence	5-year Survival	Diagnosed Late-Stage
	30.3	10%	82%	23.8	7%	84%	49.9	68%	71%
Federated States of Micronesia (FSM), combined	**Oropharynx**			**Lung**			**Cervical, Invasive**		
	Incidence	5-year Survival	Diagnosed Late-Stage	Incidence	5-year Survival	Diagnosed Late-Stage	Incidence	5-year Survival	Diagnosed Late-Stage
	15.8	49%	67%	19.9	6%	91%	22.7	46%	72%
Chuuk, FSM	**Lung**			**Liver**			**Breast**		
	Incidence	5-year Survival	Diagnosed Late-Stage	Incidence	5-year Survival	Diagnosed Late-Stage	Incidence	5-year Survival	Diagnosed Late-Stage
	13.5	9%	91%	6.2	14%	100%	9.4	31%	94%
Pohnpei, FSM	**Cervical, Invasive**			**Oropharynx**			**Breast**		
	Incidence	5-year Survival	Diagnosed Late-Stage	Incidence	5-year Survival	Diagnosed Late-Stage	Incidence	5-year Survival	Diagnosed Late-Stage
	40.7	54%	66%	25.2	47%	64%	40.6	52%	77%
Yap, FSM	**Oropharynx**			**Lung**			**Liver**		
	Incidence	5-year Survival	Diagnosed Late-Stage	Incidence	5-year Survival	Diagnosed Late-Stage	Incidence	5-year Survival	Diagnosed Late-Stage
	53	50%	71%	32.2	4%	8%	20.5	0%	73%
United States (cancer incidence per 100,000 people)	Lung	Liver	Breast	Oropharynx	Cervical, Invasive	Prostate			
	60.2	8.1	124.7	12.0	7.5	109.5			

**Table 2:** Risk factors and possible solutions for diet and nutrition, exercise and obesity, economy and infrastructure, and educational attainment related to Micronesians with respect to cancer.

Key Concept	Risk Factor	Possible Solutions
Diet and Nutrition	Increased reliance on imported canned foods Traditional fibrous starches replaced with imported and refined grains Low fruit and vegetable diets Stigmatization of cultural farming Loss of traditional fishing and agricultural practices	Create culturally informed interventions to promote sustainable local food production Government subsidized programs to promote and access traditional fishing and agricultural practices
Exercise and Obesity	High rates of child and adult obesity Sedentary lifestyle Lack of facilities and programs to promote routine exercise	Provide incentives to engage in physical exercise programs Develop resources such as gyms and health centers
Economy and Infrastructure	Lack of economic diversity contributing to stunted economic growth Low household incomes Lack of transport infrastructure Aged and underfunded clinical facilities	Diversify economy and workforce opportunities and stimulate economic growth Invest in national transportation system Modernize clinical facilities
Educational Attainment	Low educational attainment Limited English while living in the US Low health literacy	Invest in and encourage educational equity for Micronesians through leadership

**Table 3:** Risk factors and possible solutions for radiation exposures and substances related to Micronesians with respect to cancer.

Key Concept	Risk Factor	Possible Solution
Radiation Exposure	Exposure to radioactive air particles Radiation contaminated food supply Radiation-related trauma	Expand publicly available research on radiation levels in Micronesia to inform public policy Create culturally competent interventions addressing radiation-exposure trauma
Betel Nut and Substance Use	Chewing betel nut with or without tobacco Adolescents chewing betel nut Smoking tobacco Adolescent smoking and secondhand smoke exposure Heavy alcohol consumption (>6 drinks/day)	Develop clinical trials that focus on improving the cancer outcomes for patients with betel nut related oral cavity cancers Improve health literacy around betel nut and substance use in relation to cancer

**Table 4:** Summary table of cancer surveillance in the USAPI. Available resources are categorized by island. Data based on Pacific Regional Central Cancer Registry 2007–2018 report [18].

Cancer Surveillance in USAPI							
	CDC Breast and Cervical Early Detection Program	Mammography	Pap Smears	Prostate Cancer Screening (PSA)	CT On-Island	Colonoscopy	Transrectal Ultrasound
Guam	Available	Available	Available	Available	Available	Available	Available
Commonwealth of the Northern Mariana Islands	Available	Available	Available	Available	Available	Available	Unavailable
Republic of Marshall Islands	Available	Available	Available	Available	Unavailable	Available	Available
Republic of Palau	Available	Available	Available	Available	Available	Available	Unavailable
Federated States of Micronesia (FSM)							
Chuuk, FSM	Unavailable	Unavailable	Available	Unavailable	Unavailable	Unavailable	Unavailable
Kosrae, FSM	Unavailable	Unavailable	Available	Unavailable	Unavailable	Unavailable	Unavailable
Pohnpei, FSM	Unavailable	Private Provider	Available	Available	Private Provider	Unavailable	Unavailable
Yap, FSM	Unavailable	Unavailable	Available	Available	Unavailable	Available	Unavailable

**Table 5:** Summary table of available cancer diagnostic and treatment services in the USAPI. Data based on Pacific Regional Central Cancer Registry 2007–2018 report [18].

Cancer Diagnosis and Treatment								
	Pathologist	Radiologist	FNA	MRI/ PET Scan	Surgical Specialist	Chemotherapy	Radiation Therapy	Off-Island Referral System
Guam	Available	Available	Available	Available	Gen. Surg., Urologist, OB-Gyn, Oncologist, Surg. Subspecialist	Available	Available	Available
Commonwealth of the Northern Mariana Islands	Available	Available	Available	Unavailable	Gen. Surg., OB-Gyn, Oncologist, Surg. Subspecialist	Available	Unavailable	Available
Republic of Marshall Islands	Unavailable	Available	Available	Unavailable	Gen. Surg., OB-Gyn, ENT	Unavailable	Unavailable	Available
Republic of Palau	Unavailable	Unavailable	Available	Unavailable	Gen. Surg., OB-Gyn	Unavailable	Unavailable	Available
**Federated States of Micronesia (FSM)**								
Chuuk, FSM	Unavailable	Unavailable	Unavailable	Unavailable	Gen. Surg., OB-Gyn, Surg. Subspecialist	Unavailable	Unavailable	Available
Korae, FSM	Unavailable	Unavailable	Unavailable	Unavailable	Gen. Surg., OB-Gyn	Unavailable	Unavailable	Available
Pohnpei, FSM	Unavailable	Unavailable	Available	Unavailable	Gen. Surg., OB-Gyn, Ortho	Available	Unavailable	Available
Yap, FSM	Unavailable	Unavailable	Available	Unavailable	Gen. Surg., OB-Gyn	Available	Unavailable	Available

**Table 6:** Risk factors and possible solutions for healthcare access, health insurance, and perceptions of cancer care related to Micronesians with respect to cancer. Abbreviation: COFA—Compact of Free Association.

Key Concept	Risk Factor	Possible Solutions
Healthcare Access	Extremely remote, water-bound island locations with limited healthcare resources Inaccessible intra- and inter-island transportation for goods, patients, and healthcare workers Scarcity or complete lack of physician specialists	Strengthen transportation access, particularly around healthcare systems Increase recruitment of and accessibility to physician specialists, particularly oncologists through telehealth technologies
Health Insurance	Underinsurance and lapses in insurance coverage with COFA Prior exemption of Micronesian migrants from Medicaid expansion	Expand research on current Micronesian COFA migrant insurance coverage
Perceptions of Cancer Care	Low cancer screening rates Trust in physicians lower than spiritual leaders Lack of cancer screening access in populations that highly desire screening	Improve trust in physicians with community engagement programs Increase access to cancer screening Strengthen the local physician workforce pipeline

**Table 7:** Risk factors and possible solutions for healthcare access, health insurance, and perceptions of cancer care related to Micronesians with respect to cancer.

Key Concept	Risk Factor	Possible Solutions
Climate Change	Sea level rise impacting low-lying islands and atolls Wetland erosion with severe weather events threaten critical facilities Food source disruptions Potential increase in atmospheric carcinogen exposure	Develop data systems to monitor changes in Micronesian climate correlated with health status Create effective health-protective interventions for high-risk islands Develop protocols for climate resilient healthcare systems within hospitals throughout Micronesia

**Table 8:** Risk factors and possible solutions for physician workforce diversity related to Micronesians with respect to cancer.

Key Concept	Risk Factor	Possible Solutions
Physician Workforce Diversity	Underrepresentation of indigenous Pacific Islander physicians “Brain-drain” causing lapses in continuity of care Lack of specialty care, including oncology services, throughout Micronesia	Improve Pacific Islander physician representation to maintain local talent with a vested interest in the care of Micronesian communities Create lasting pipeline programs for aspiring prospective physicians with strong Micronesian community ties
